# Automatic classification of cervical cancer from cytological images by using convolutional neural network

**DOI:** 10.1042/BSR20181769

**Published:** 2018-11-28

**Authors:** Miao Wu, Chuanbo Yan, Huiqiang Liu, Qian Liu, Yi Yin

**Affiliations:** 1 College of Medical Engineering and Technology, Xinjiang Medical University, Urumqi 830011, China; 2 Basic Medical College, Xinjiang Medical University, Urumqi 830011, China

**Keywords:** Cervical Cancer, Classification, Cytological Images, Deep Convolutional Neural Networks, Subtypes

## Abstract

Cervical cancer (CC) is one of the most common gynecologic malignancies in the world. The incidence and mortality keep high in some remote and poor medical condition regions in China. In order to improve the current situation and promote the pathologists’ diagnostic accuracy of CC in such regions, we tried to propose an intelligent and efficient classification model for CC based on convolutional neural network (CNN) with relatively simple architecture compared with others. The model was trained and tested by two groups of image datasets, respectively, which were original image group with a volume of 3012 datasets and augmented image group with a volume of 108432 datasets. Each group has a number of fixed-size RGB images (227*227) of keratinizing squamous, non-keratinizing squamous, and basaloid squamous. The method of three-folder cross-validation was applied to the model. And the classification accuracy of the models, overall, 93.33% for original image group and 89.48% for augmented image group. The improvement of 3.85% has been achieved by using augmented images as input data for the model. The results got from paired-samples *t*test indicated that two models’ classification accuracy has a significant difference (*P*<0.05). The developed scheme we proposed was useful for classifying CCs from cytological images and the model can be served as a pathologist assistance to improve the doctor’s diagnostic level of CC, which has a great meaning and huge potential application in poor medical condition areas in China.

## Introduction

Cervical cancer (CC) remains one of the leading causes of cancer-related deaths in women worldwide [1], with 80% of the cases occurring in developing countries [2]. And China is one of them with high CC incidence and mortality rates [3]. A study indicated that the crude incidence rates of CC in Chinese rural and urban areas were 11.87/100000 and 11.98/100000, respectively in 2007–2008, meanwhile, the crude mortality rates were 2.19/100000 and 3.20/100000 [4]. Moreover, the incidence of this cancer in young Chinese women (≤30 years old) is increasing by 2–3% yearly [5]. In some remote districts like Xinjiang Uyghur Autonomous Region in Northwest China, which has poor medical conditions like insufficient healthcare accessibility and qualified medical staff, CC incidence and mortality are even higher. It has already been an extremely important public health issue in Xinjiang area. The data from Chinese Health Statistics Yearbook published in recent years indicated that the overall level of Xinjiang public medical and health conditions, such as healthcare facilities, health funds, health technicians, medical service etc. are lower than the national average. Due to the historical reasons and unbalanced development in China, the situation of medical and health condition in Southern Xinjiang is even worse, which is urgently needed to improve.

The contemporary artificial intelligence techniques such as machine learning applications were widely used in medical health field in recent years and achieved the certain success [6–14]. It can serve as an excellent assistance for disease diagnosis, prognosis as well as treatment, and could greatly enhance the work of medical experts and ultimately to improve the efficiency and quality of medical care, which have important meanings to improve medical level especially for those poor medical resource areas.

During the past few years, an important machine learning algorithm named deep convolutional neural network (DCNN) has a very prominent achievement on medical image classification and tremendous progress has been made in this area [15–20]. Dorj et al. [15] proposed an intelligent and rapid classification system of skin cancer using contemporary, highly efficient DCNN, whose overall value of average accuracy is greater than 90%. Teramoto et al. [16] developed an automated classification scheme for lung cancers presented in microscopic images using DCNN and the results showed that approximately 71% of images were correctly classified, which is at par with the accuracy of cytotechnologists and pathologists. Tsehay et al. [17] developed a computer-aided detection (CAD) system based on DCNN, which can automatically detect lesions on multi-parametric MRI. Wahab et al. [18] presented a two-phase model to mitigate the class biasness issue while classifying mitotic and non-mitotic nuclei in breast cancer histopathology images through DCNN. Sharma et al. [19] explored DCNN for computer-aided classification on histopathological images of gastric carcinoma, with an overall classification accuracy of 0.699 for cancer classification and 0.8144 for necrosis detection. Khosravi et al. [20] utilized several computational methods based on convolutional neural networks (CNN) and built a stand-alone pipeline to classify different histopathology images across different types of cancers effectively.

Our study is inspired by the current medical and disease situation in Xinjiang where the qualified medical staff is insufficient and the incidence and mortality of CC are high. Moreover, precise and rapid diagnosis is an important prerequisite for further treatment. So, we are trying to develop computer-aided diagnosis (CADx) [21] schemes with the main purpose of automatically classifying the different CC types from pathological images so as to enhance the pathologist’s work efficiency.

By reading the extensive literature, we found researchers around the world had carried out various studies on CADx on CC recent years. Zhang et al. [22] proposed a method based on CNNs to directly classify SINGLE cervical cell into normal and abnormal from image patches centered on the nucleus centroid. Xu et al. [23] designed a deep learning framework for cervical dysplasia diagnosis by leveraging multimodal information such as non-image data and image data. Devi et al. [24] discussed the different types of methods used for the detection of CC based on neural networks. Taha et al. [25] proposed a deep learning approach for detecting cervix cancer from pap-smear images, employing pre-trained CNN architecture as a feature extractor and using the output features as input to train a Support Vector Machine Classifier. However, the methods mentioned above did not meet our requirements as they needed strict prerequisites. For example, a nucleus center was pre-required for applying the method of Zhang et al. [22], and was obtained from the ground truth segmentation. However, screening of abnormal cells within a given field-of-view required automated detection of nucleus centers. The method proposed by Xu et al. [23] needed multimodal information which was not easily to collect completely in practical such as cervigram, data of Pap test and HPV test etc. Devi et al. [24] only discussed the methods in theory which were not implemented. Taha et al. [25] methods also needed pre-required work like accurate cell image segmentation which remained a tough problem especially when the images contained adherent cells.

In the present paper, we proposed an easy and practical method for the classification of CC from cytological images without handcraft feature extraction or precise cell image segmentation work. Instead, we directly used the cytological images containing several CC cells as input to the pipeline. Actually, our previous work [9] demonstrated a DCNN system based on AlexNet to automatically classify the different types of ovarian cancers from cytological images with preferable classification accuracy. Based on the experience of previous research, we tried to construct and verified the model whether it can correctly classify the CC images as well.

Squamous cell carcinoma (SCC) is the predominant histological type accounting for three-fourths of all CCs. Adenocarcinoma and adenosquamous cell carcinoma represent 10–15%, and other or unspecified histology represent the remaining 10–15% [26]. According to WHO Histological Classification Of Tumors Of The Uterine Cervix [27], the current subtypes of SCC are as follows: Keratinizing, Non-keratinizing, Basaloid, Verrucous, Warty, Papillary, Squamotransitional, Lymphoepithelioma-like. While adenocarcinomas’ subtypes are endocervical adenocarcinoma, mucinous carcinoma, villoglandular carcinoma, endometrioid carcinoma, clear cell carcinoma, serous carcinoma, mesonephric carcinoma, adenocarcinoma admixed with neuroendocrine carcinoma. As our previous research has demonstrated that the DCNN model has the ability to distinguish the different subtypes, cytological images from ovarian adenocarcinomas including serous, mucinous, endometrioid and clear cell, which have similar morphology to cervical adenocarcinomas, we believe that the DCNN model can also distinguish adenocarcinomas subtypes of CC. And in the present study, we focussed on augmenting image datasets and constructing the DCNN model to recognize SCC subtypes images of CC. To the best of our knowledge, DCNN have not been applied to the classification works on SCC of CC from cytological images yet.

## Materials and methods

### Image dataset

#### Image acquisition

**Figure 1:**
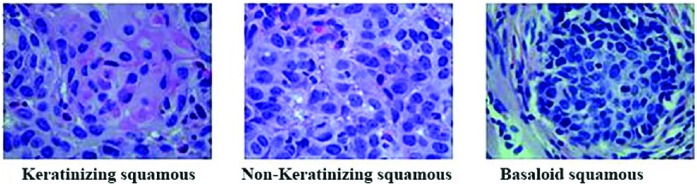
Cytological image samples of CC in 400× magnification (2048*1536 pixels)

Seventy-nine (79 specimens in total, 31 Keratinizing squamous, 27 Non-keratinizing squamous, 21 Basaloid squamous) Hematoxylin–Eosin (H&E) stained 3 µm-thick tissue sections of CC from years 2003 to 2015 were collected from the First Affiliated Hospital of Xinjiang Medical University for subtype classification. And every H&E slide was histologically confirmed. In order to get more image datasets for the later research work, we captured several images (5–12 pieces) manually from different regions of each H&E stained tissue sections by a digital still camera attached to a microscope (Model: Leica, Brand: DM300, Place of origin: Germany) with 40× objective lens, while keeping their orientation invariable. For the purpose of getting qualified image data, we tried to select the clear images for the experiment, which were fulfilled with unique subtype cells of CC. Most of them had prominent clinic pathological features, such as tissue morphologies and colors etc. Figure 1 showed the different subtype image samples of CC.

#### Image preprocessing

**Figure 2:**
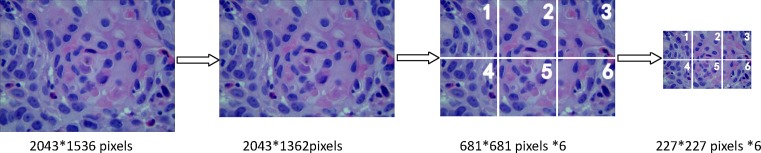
Images preprocessing for classification of CC by DCNN

Thus, a total of 502 pathological images containing three different subtypes were collected in JPG format. The initial matrix size of each JPG image was 2048*1536 pixels. For the needs of follow-up research, we did the following preprocessing work for each image. First, the initial images were cropped to the size of 2043*1362 pixels. Second, we divided each of 2043*1362 pixels image into six square patches with the same size which was 681*681 pixels. Third, all the square patches were resized into 227*227 pixels and named as original images. At last, we got 3012 original images. Figure 2 showed the image preprocessing work.

The study was reviewed and approved by an institutional review board (ethics committee). With the main purpose of protecting the patients’ privacy, all the H&E-stained tissue sections were anonymous and the information of patients’ identification was kept secret.

#### Image augmentation

**Figure 3:**
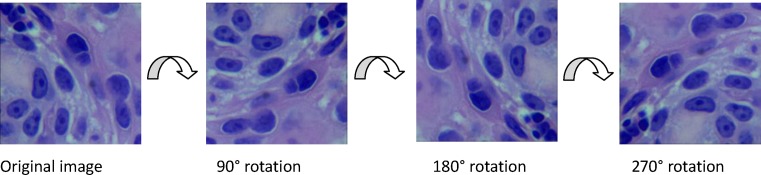
Image rotation

**Figure 4:**
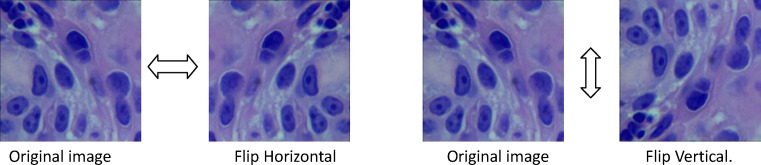
Image flipping

**Figure 5:**
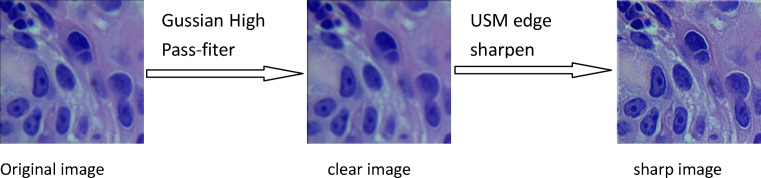
Image enhancement

**Figure 6:**
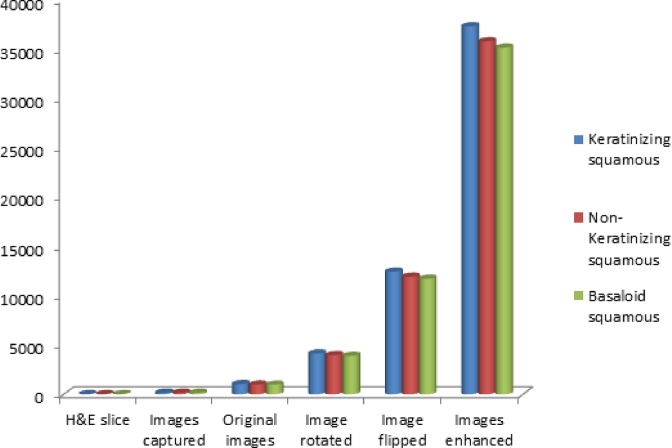
Image quantities of three distinct CC subtypes at different stages

Deep learning, such as DCNNs, is a recently developed method that yields very successful results in image classification. DCNNs, which have a high number of parameters, require a large amount of data to avoid overfitting during training [28]. Because the sample size in our study was limited, the image datasets for training were augmented by image rotation, image flipping, and image enhancement technology, after which the sample size were greatly expended. Figure 3 showed the process of image rotation. Figure 4 showed image flipping. A Gaussian High Pass-filter with kernel size = 4*4 for image denoting and An Unsharp Mask (USM sharpen) with kernel size = 5*5, threshold value = 20 color gradation, quantities = 170% for edge sharping, were applied to enhancement images clarity and sharpness. Figure 5 showed the process of image enhancement. The above manipulations made a sample size 36-times the original sample size. Table 1 and Figure 6 showed image quantities of three distinct CC subtypes at different stages, which indicated that the sample size of the subtypes are almost the same. Then we trained and validated the DCNN model with original image data and augmented image data separately. The results were compared and explained.

**Table 1:** Image quantities of three distinct subtypes at different stages

	Keratinizing squamous	Non-keratinizing squamous	Basaloid squamous
H&E slice (3-µm-thick)	31	25	23
Images captured (Size: 2048*1536 pixel)	173	166	163
Original images (Size: 227*227 pixel)	1038	996	978
Images rotated (Size: 227*227 pixel)	4152	3984	3912
Images flipped (Size: 227*227 pixel)	12456	11952	11736
Images enhanced (Size: 227*227 pixel)	37368	35856	35208

### DCNNs architecture

**Figure 7:**
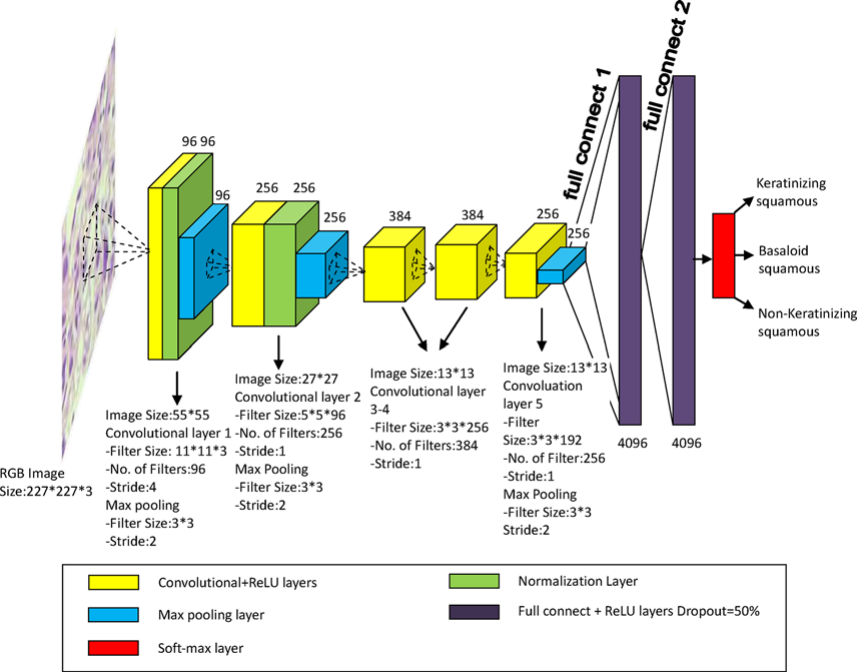
The DCNN architecture and annotation for pathological image classification of CC

DCNN is a biologically inspired class of deep learning models that has achieved excellent performance on visual and speech recognition problems [29]. A typical DCNN involves four types of layers: convolutional, activation, pooling, and fully connected layers [30]. Our DCNN model was constructed based on a very famous DCNN named AlexNet, which was first proposed by Krizhevsky et al. [31] in the 2012 ImageNet Large Scale Visual Recognition Challenge (ILSVRC-2012). Compared with the other structure–complex DCNN architectures (e.g. GoogLeNet [32], VGG et al. [33]), AlexNet is a structure-simple and highly efficient DCNN, which is easy to train and optimize. It mainly consisted of cascaded stages, namely, convolution layers, pooling layers, rectified linear unit (ReLU) layers and fully connected layers. Figure 7 showed the architecture and annotation of our DCNN model for CC pathological image classification.

From Figure 7 we can see RGB image with size 227*227 was the input part of DCNN, meanwhile the output part was probabilities of three CC subtypes, which were calculated by a soft-max function. Our DCNN model was composed of five convolutional layers, each of which (yellow cube) was followed by ReLU. They were usually used to extract image features automatically. And three max pooling layers (blue cube) with filter size of 3*3 and stride of 2 were inserted between the convolutional layers to decrease the spatial size of input images and the amount of tunable parameters. Two fully connected layers (purple cube) were applied at the end of model, which consisted of all previous 4096 neurone connections. It normally promotes the reduction in spatial information [34]. Dropout is a regularization technique for reducing overfitting in neural networks by preventing complex co-adaptations on training data. It is a very efficient way to perform model averaging with neural networks [35]. And the dropout rate in our model was 50%.

### Transfer learning and fine-tuning

Deep learning often requires large datasets to train the networks, which are lacking in the medical domain especially for CC images, while transfer learning proved to be an efficient way to deal with such problems [37]. Bengio [38] demonstrated that transfer learning in CNN could be achieved first by training a CNN on a domain with a large amount of data, and then re-training that CNN on a small and different domain via fine-tuning its weights. Hoochang et al. [39,40] showed that transfer learning can be beneficial even in two very different domains (natural and medical). The advantages of transfer learning even extended beyond the limited data issue, where it was proven to be an effective initialization technique for building robust deep learning models [41,42]. In our study, we first initialized the model with pre-training on ImageNet dataset [43] and then duplicated it to create two same models. One model was fine-tuned on the original image sets and the other one was fine-tuned on the augmented image sets. Both of the convolutional layers in the models were initialized with pre-trained weights and learning rate multipliers of 0.1 were applied.

### The hardware and software for performing classification works by DCNN

Traditional calculation work usually performed by Central Processing Unit (CPU) optimized for single-threaded performance. However, applying CNNs to large images is much computationally expensive (work) because of large amount of computation scales linearly with the number of image pixels [36]. Thus, we used multithreaded GPU to train the model as it achieved high throughput by running thousands of threads in parallel. Our study was carried out with the help of GeForce GTX TITAN X with 12 GB of Random Access Memory (RAM), Intel® Core™ i7-7500U Processor (4 M Cache, up to 3.50 GHz ), and 4 GB DDR SDRAM. The DCNN was built and trained by the deep learning freamwork-Caffe package under the Ubuntu 16.04 operation system.

## Results

**Figure 8:**
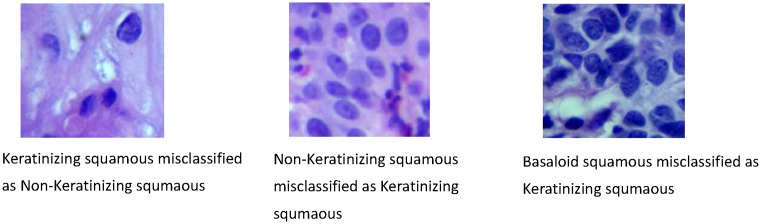
Misclassified samples of CCs by the DCNN

Two classification models that had the same architecture as shown in Figure 7 were trained and tested by two groups of image dataset respectively, which were original image group with a volume of 3012 datasets and augmented image group with a volume of 108432 datasets. For each image group, images were divided into three sets including training, validation, and test sets. For this purpose, 70% of all images were allocated to the training sets and the remaining images were devoted to validation sets (15%) and test sets (15%). By using our models, the image features were extracted automatically and the images were classified into three groups, i.e. keratinizing squamous, non-keratinizing squamous, and basaloid squamous. The classification accuracy was calculated by means of three-folder cross-validation method, as all the image datasets were randomly divided into three same-size groups. Table 2 showed the number of image datasets in each folder. Table 3 showed the result of classification accuracy, overall, 89.48% for original data and 93.33% for augmented data. Table 4 was the confusion matrix of CC subtype classification results for original images and augmented data, respectively. It can be seen that Keratinizing squamous and Non-keratinizing squamous were often misclassified. Figure 8 showed the misclassified image samples of CC by the models.

**Table 2:** Number of image datasets in every group for three-fold cross-validation test

	Keratinizing squamous		Non-keratinizing squamous		Basaloid squamous	
	Original	Augmented	Original	Augmented	Original	Augmented
Image datasets	346	12456	332	11952	97	11736

**Table 3:** Classification accuracy of two models

	Model trained by original dataset	Model trained by augmented dataset
Keratinizing squamous	88.74%	94.41%
Non-keratinizing squamous	89.56%	92.03%
Basaloid squamous	90.14%	93.54%
Average	89.48%	93.33%

**Table 4:** Confusion matrix of CC subtype classification results

	Keratinizing squamous		Non-keratinizing squamous		Basaloid squamous	
	Original	Augmented	Original	Augmented	Original	Augmented
Keratinizing squamous	88.74%	94.41%	5.33%	3.62%	5.93%	1.97%
Non-keratinizing squamous	6.97%	5.38%	89.56%	92.03%	3.47%	2.59%
Basaloid squamous	6.38%	3.72%	3.48%	2.74%	90.14%	93.54%

## Discussion

In general, more than 93.33% CC images were classified correctly, which is a satisfactory result compared with other classification models. It indicates that our DCNN model has a strong ability to recognize SCC subtypes images of CC. Most correctly classified images had a certain number of cells in it with notable pathological features, such as cell morphology, tissue color, cell distribution etc., while misclassified images had poor features. The results showed images of keratinizing squamous and non-keratinizing squamous are often misclassified. We thought this might be caused by the process of image splitting. An image obtained by the digital still camera was split automatically into six parts without pathologists’ confirmation, some of which might have less pathological features such as insufficient cancer cells and intercellular substance etc. Furth more, few images (size: 2048*1536 pixel) may even have both keratinizing squamous part and non-keratinizing squamous part before splitting. However, in our study we simply treat the six parts (size: 227*227 pixel) generated from them as ONE subtype images, which could easily lead to training problems and misclassification. Due to the large number of splitting images (3012), it was difficult to confirm every image manually by few pathologists, which required us to get clear images as fulfilled with one subtype cells and tissues as possible in the process of image acquisition. Another point worth mentioning is that the classification accuracy of model trained by augmented data was 93.33%, which was 3.85% greater than the result of model trained by original data (89.48%). A paired samples *t*test was performed base on the results of three-folder cross validation, which indicated two models’ classification accuracy has a significant difference (*P-*value less than 0.05). As two models with the same architecture were trained, tested under the same environment, we firmly believed that the significant difference is mainly caused by the process of image augmentation (image rotation, image flipping, image denoising, and image sharpness).

Ultimately, three facts have been proved through our study: first, the DCNN model we proposed had a strong ability to recognize the different cytological images of CCs subtype. Second, the classification accuracy of the model can be improved by image augmentation. Last but not the least, our method is easy to perform and has practical meanings for CADx of CC. However, in order to improve the results our further investigations will mainly aimed at improving the classification accuracy by means of using other DCNN models such as GoogLeNet, VGG etc. and new methods of data augmentations.

## Conclusion

We have investigated the utility of the DCNN for classifying of CC subtypes on cytological image received from H&E-stained tissue sections. By increasing the training sample size with help of image process (including image rotation, flipping, enhancement), classification accuracy of the model has been improved by 3.85% and a satisfactory classification accuracy of 93.33% was achieved. The model and scheme we proposed in the present study can serve as a pathologist’s assistance to improve the doctor’s diagnostic level of CC for those regions where the medical condition is poor and the pathologist is insufficient.

## Abbreviations

CAD: computer-aided detection
CADx: computer-aided diagnosis
CC: cervical cancer
CNN: convolutional neural network
DCNN: deep CNN
H&E: Hematoxylin–Eosin
ReLU: rectified linear unit
SCC: squamous cell carcinoma
