# Correction: Automatic classification of cervical cancer from cytological images by using convolutional neural network

**DOI:** 10.1042/BSR-20181769_COR

**Published:** 2019-04-02

***Bioscience Reports* (2018) 38(6), BSR20181769; https://doi.org/10.1042/BSR20181769**

The authors have made an error in the abstract of their published article. The sentence “93.33% for original image group and 89.48% for augmented image group” should read “89.48% for original image group and 93.33% for augmented image group”.

